# Qualification of the Dental Student

**Published:** 1878-10

**Authors:** 


### EDITORIAL, ETC.

" I desire to attend lectures at your Dental College this winter, and write to know concerning the terms, requirements &c." And this correspondent goes on to relate how long his pupilage has been, the standing, &c., of his preceptor, the qualifications of hand he (the student) is thought to possess; and wishes to know if he cannot " graduate in one session," as he hears others have done.

It is not hard to decide what answer to make this student, less difficult perhaps than if he had sought a personal interview. His letter betrays that however well trained he may be in the routine of office practice, his mental training and general education is sadly deficient, so deficient indeed as to make it sure that if he were in any school it should be one in which the more common English branches are taught. He is anxious to obtain a diploma which should entitle him to entrance into the best society, and which places him in competition, in the location he will probably select as an eligible field, with polished and cultivated men, whose peers in society he cannot hope to be, except through years of the closest study and application. As well might an ignorant and coarse country bumpkin, marry a refined and elegant lady and expect to feel comfortable and reflect credit upon her choice, as for a man with not sufficient mental training to qualify him for creditable comparision with dentists, expect to do well in the race with those thus equipped.

But do Dental Colleges insist upon certain previous qualifications and preparation on the part of those applying for tickets? asks one to whom this letter of enquiry is shown. No certain standard can be set which would be perfectly just, we answer, for obvious reasons. But that *some* requirements of the sort should be possessed, is evident, for the protection not only of a noble and elevated profession which should not be hampered by the ignorant any more than by the pretender, but for the protection of the teachers themselves. Those holding professorships in Dental Colleges do not undertake, nor could it be expected of them, to teach the plainer English branches, (the older provision that the student should possess some familiarity with Latin and Greek seems obsolete now;) and if students cannot understand the ordinary verbiage of the lecturer, simplified as he strives to make it, it is only just to the teacher that the student should possess the preliminary training which would place him on a plane with the teacher's language. It is a matter of common remark among students that the lecturer overshoots his hearers. This is not because the former is abstruse, but because the latter are ignorant of the medium of communication between them and the speaker.

It is due the profession, the schools, and the student himself for his own protection, that he should know something—the more the better,—something which will entitle him to the respect of his associates and the public; that he should at least know enough to comprehend the ordinary speech of the lecturer, and to some extent be a master of the technicalities of ordinary scientific language; that the lecturer should not be required to lay these foundations for him, and that he represent creditably in society the honorable profession of which he is a member. Less than this should not in fairness be expected of a man who is to deal with his humanly organized fellow-beings in one of the most perplexing and pains-taking, as well as sympathetic of callings.

So much for the thought that arises from a perusal of his letter, which is respectful in its tone, and displays an ardent desire to be something. And so impressed are we with this that we write to him, to the effect that, while we fear he is not in a sufficiently advanced position to make the most of a course of lectures, yet he had best have a personal interview with some one connected with the college, that he may show as well what he does know, as his letter betrays his want of knowledge. The result of this interview shows that his preceptor, though a man standing high in his profession, with a large practice, has had such ill-defined notions of the relations between himself and his pupil, as to limit his teaching to the ordinary details of laboratory work, with only an occasional opportunity on the part of the student to see operations, and with no attempt whatever to lay broad and deep the foundations on which the scientific study of dentistry must stand. The study of chemistry, of anatomy, of physiology &c., has not only not been attempted, but their necessity not even suggested ; the main idea inculcated being that the dentist should be a man of *work*, not thought, and that the mechanism of the profession is its all—a *practical* profession.

We can only advise this young man. The schools are open, and he has a right to enter. But that such students beset the path of the teacher with perplexing difficulties is evident indeed. Lectures must be simplified for him and technicalities avoided, and the course of study for those who are better qualified made trite and commonplace.

Where is the fault? With the Colleges? Hardly. With the Student? No. With the Preceptor? Undoubtedly.

